# Acute mercury poisoning presenting as fever of unknown origin in an adult woman: a case report

**DOI:** 10.1186/1752-1947-8-266

**Published:** 2014-08-01

**Authors:** Gonul Cicek-Senturk, Fatma Aybala Altay, Aysegul Ulu-Kilic, Yunus Gurbuz, Ediz Tutuncu, Irfan Sencan

**Affiliations:** 1 Department of Infectious Diseases and Clinical Microbiology, Dişkapi Yildirim Beyazit, Training and Research Hospital, Ankara, Turkey; 2 Department of Infectious Diseases and Clinical Microbiology, Erciyes University School of Medicine, Kayseri, Turkey

**Keywords:** Fever of unknown origin, Mercury, Metal fume fever, Rash, Lymphadenopathy

## Abstract

**Introduction:**

Mercury intoxication may present in a wide range of clinical forms from a simple disease to fatal poisoning. This article presents a case of acute mercury poisoning, a rare condition that presents challenges for diagnosis with fever of unknown origin.

**Case presentation:**

A 52-year-old Caucasian woman was admitted to the hospital with high fever, sore throat, a rash over her entire body, itching, nausea, and extensive muscle pain. She had cervical, bilateral axillary and mediastinal lymphadenopathies. We learned that her son and husband had similar symptoms. After excluding infectious pathologies, autoimmune diseases and malignancy were investigated. Multiple organs of our patient were involved and her fever persisted at the fourth week of admission. A repeat medical history elicited that her son had brought mercury home from school and put it on the hot stove, and the family had been exposed to the fumes for a long period of time. Our patient’s serum and urine mercury levels were high. She was diagnosed with mercury poisoning and treated accordingly.

**Conclusions:**

Mercury vapor is a colourless and odorless substance. Therefore, patients with various unexplained symptoms and clinical conditions should be questioned about possible exposure to mercury.

## Introduction

Disorders that result in classical fever of unknown origin (FUO) fall into one of five categories: infections, neoplasms, connective tissue diseases, miscellaneous disorders, and undiagnosed illnesses. Metal fume fever is included in miscellaneous disorders [1]. The fumes or gases are a form of several metals, for example cadmium (Cd), manganese (Mn), mercury (Hg), and zinc chloride (ZnCl2). Metal fume fever, which may occur after inhaling the metal fumes, is a poorly understood influenza-like reaction [2]. The clinical effects of mercury poisoning depend on the form and the route of entry to the organism. Acute inhalation of high vapor concentrations, usually from heated elemental mercury, can result in acute lung injury with symptoms of cough, sore throat, shortness of breath, and chest pain. Other symptoms include fever, erythematous rash, itching, chills, gastrointestinal complaints, metallic taste, headache, and weakness [3].

## Case presentation

A 52-year-old Caucasian woman presented to a healthcare center with high fever, sore throat, itchy rash, generalized muscle pain, and nausea, which had continued for 15 days. The patient’s husband and son had similar complaints over the same period. She was administered Penicillin G, antihistaminic and antipyretic drugs. Because her condition did not improve, she was referred to a dermatology clinic with the suspicion of urticarial vasculitis. She received antihistaminic and depot steroid treatment there and a consultation with the infectious diseases department was requested. Her body temperature was 39°C (centigrade), her pulse rate was 96 beats/minute, and respiratory rate was 22 breaths/minute at first examination. She had a generalized maculopapular rash spread over her entire body, even the palms of her hands and the soles of her feet. Our patient had bilateral cervical and axillary painful and mobile lymphadenopathies.

Her laboratory test results were as follows; serum urea: 151mg/dL (normal range: 10 to 50mg/dL), serum creatinine: 2.6mg/dL (normal range: 0.6 to 1.1mg/dL), lactate dehydrogenase (LDH): 608U/L (normal range: 207 to 414U/L), total protein: 5.8g/dL (normal range: 6.4 to 8.3g/dL), albumin: 2.7g/dL (normal range: 3.8 to 5.1g/dL), white blood cell (WBC) count: 22,300/μL (normal range: 4300 to 10,300/μL), red blood cell (RBC) count: 4.02×106/μL (normal range: 4.38 to 5.75×106/μL), hemoglobin: 12.4g/dL (normal range: 13.6 to 17.2g/dL), erythrocyte sedimentation rate (ESR): 45mm/h, C-reactive protein (CRP): 52mg/L (normal range: 0 to 5.0mg/L).

Abdominal ultrasonography (USG) revealed that hepatomegaly was present. All other intra-abdominal structures were normal. Her cervical USG revealed a large number of reactive lymphadenopathies at submandibular, submental, posterior cervical, upper and lower jugular regions bilaterally and at the right and left submandibular regions. Some of them were conglomerated. There were also several reactive lymphadenopathies at both her axillary regions, which were observed on the axillary ultrasonography. A thoracic computed tomography (CT) scan was performed because our patient’s chest radiography revealed several uncertain nodular lesions. The thoracic CT scan presented widespread areas of air trapping. Bilateral multiple lymphadenopathies were present in the aortic pulmonary, prevascular, precarinal, paratracheal, subcarinal and left supraclavicular regions.

Levofloxacin treatment was initiated empirically at the dermatology clinic. On the fifth day, this treatment was discontinued and our patient was transferred to the infectious diseases clinic because there was no improvement in her signs and symptoms.

Measles, Rubella, *Epstein-Barr virus* (EBV), *Cytomegalovirus* (CMV), Toxoplasma immunglobulin (Ig) M tests were reported to be negative. The tests for Ricketsiosis, Coxiellosis, Brucellosis and Tularemia were reported to be negative. The autoimmune markers were studied to exclude a rheumatologic disease and found to be negative.

**Figure 1:**
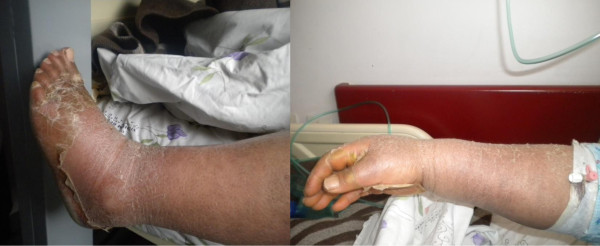
Desquamation of the hands and feet of our patient.

On the twelfth day of admission, our patient’s WBC count increased to 35,000μL and her hemoglobin decreased to 10.6g/dL (normal range: 13.6 to 17.2g/dL). Therefore, our patient was referred to the hematologists. A total of 45% polymorphonuclear leukocytes (PMN) (50% band form), 40% lymphomonositer cells (50% atypical lymphocytes), and 11% eosinophils were identified in her peripheral blood smear. Dysplasia was also present in neutrophils. A bone marrow biopsy and tests were performed, yielding no positive results.Desquamation spread over her entire body (Figure 1), an evident erythematous rash on her palms and soles, dyspnea, chest pain and hypertension developed in the following days. Kawasaki syndrome was suspected. Our patient consulted with a chest diseases specialist and a cardiologist. An electrocardiogram and echocardiogram were performed and no cardiologic cause was found for her symptoms. Bronchodilator and antihypertensive agents were added to the treatment.

After insistent efforts of repeated medical history taking, we learned that the patient’s son had brought mercury home from school in a bottle and had spilled it on the hot stove. All of the family members (mother, father and son) were exposed to this environment of dense mercury fumes for a long period of time. Because mercury fumes are colorless and odorless, they were unable to detect it. Approximately 10 hours after this event, our patient’s husband developed dyspnea, hypertension, tachycardia, high fever and sore throat. He was admitted to the healthcare institution with these complaints. A physical examination of the patient revealed no pathological findings other than hypertension (tension arterielle (TA): 160/100mmHg) and erythema in the legs. His laboratory test results showed leucocytosis (WBC count: 13,200/μL (normal range: 4300 to 10300/μL)). Other laboratory findings were considered normal. His complaints improved with the supportive treatment administrated at the respiratory diseases clinic of the hospital. He was discharged after eight days of hospitalization, but a personality disorder developed in the following days.

The 14-year-old son suffered from high fever, sore throat, skin rash, diarrhea, and dyspnea. He was admitted to the pediatric hospital with these complaints. A physical examination revealed the following: fever: 39°C, TA: 110/50mmHg, respiratory rate: 20 breaths/minute, pulse: 104 beats/minute. A generalized maculopapular rash was spread over the patient’s entire body, and he was identified with hyperemia in the conjunctivas and cervical and bilateral axillary lymphadenopathy. Rhoncuses were determined in the lungs by oscultation. No abnormal laboratory findings, other than leucocytosis (WBC count: 11,100/μL (normal range: 4300 to 10,300/μL)) were identified. The patient was initially given empirical doxycycline and bronchodilator therapy based on the family history. A thoracic CT scan was performed. Consolidation areas containing air bronchograms were detected in the paracardiac region in the right lung middle zone. Ceftriaxone and clarithromycin were included in his treatment. The tests performed revealed no findings of infection. Material was submitted to the reference laboratory for blood and urine mercury level tests upon persistent fever on day 10 of hospitalization.

The son’s serum and urine mercury levels were analyzed using the inductively coupled plasma - mass spectrometer (ICP-MS) method in the reference laboratory. His serum mercury level was 130μg/L (normal range: 0.6 to 59μg/L), and his urine mercury level was 31μg/L (normal range: 0.1 to 20μg/L). He received chelating therapy after high levels of mercury were identified in his blood. Therefore, our patient’s serum and urine mercury levels were analyzed using the ICP-MS method in the reference laboratory. Her serum mercury level was 207μg/L (normal range: 0.6 to 59μg/L), and her urine mercury level was 524μg/L (normal range: 0.1 to 20μg/L).

The diagnosis of mercury intoxication was also corroborated by the consulted toxicologist. The toxicologist recommended the administration of penicillamine instead of dimercaprol as our patient recovered from the acute period. A dosage of 300mg of penicillamine every six hours was administered for seven days. Her clinical and laboratory test results were significantly improved by the treatment. No side effects of penicillamine were seen and our patient was discharged.

Her husband’s serum mercury level was 197μg/L (normal range 0.6 to 59μg/L), and his urine mercury level was 412μg/L (normal range 0.1 to 20μg/L). Therefore, he received the same treatment as his wife.

## Discussion

As a vapor, elemental mercury is absorbed rapidly through the lungs, reaching the blood and entering the brain. A clinical picture, which can be divided into three phases, evolves. The initial phase manifests itself as metal fume fever, the intermediate phase can be defined as the period during which severe, multiorgan symptoms of the central nervous, respiratory tract, gastrointestinal and urological systems are reported, and the late phase can be described as the period when the central nervous symptoms persist and other organ system complaints are resolved. The description of the earliest symptoms by the patients is consistent with metal fume fever, a syndrome commonly confused with a viral etiology, as was also true for these patients [4]. Our patient had also high fever, lymphadenopathy, and a diffuse maculopapular rash initially. Her liver and kidney function tests were elevated; she had dyspnea, tachycardia and chest pain during the following days.

The exact pathogenesis of metal fume fever is poorly understood. In some instances, allergic mechanisms may be involved [2]. Our case confirms mechanisms of hypersensitivity because she was diagnosed with urticarial vacuities initially and had eosinophilia in her blood smear. Because either our patient or the other family members had similar signs such as fever, diffuse skin rash and multiple lymphadenopathies predominantly, an infectious process was considered initially. Viral, bacterial infections, autoimmune diseases, malignancies and Kawasaki syndrome were evaluated in the differential diagnosis, but there were no findings to confirm these conditions.

Kawasaki syndrome is rarely seen in the adult population since it is primarily a disease of childhood. It can be seen as sporadic cases and in mini-epidemics [5]. It was reported previously that mercury intoxication had presented with elevated urine mercury levels compared to those of the matched controls [6].

Acrodinia (‘pink disease’) is a unique syndrome that can occur in children from chronic exposure to elemental mercury. Only a certain subset of exposed patients is affected, suggesting that it represents an idiosyncratic hypersensitivity response. Clinical manifestations include extremity pain, red face, red hands and feet, skin rash, gingivitis, desquamation, tachycardia, hypertension, photophobia and irritability [3,7]. Our patient had rash, significantly erythematous on her soles and palms; and developed desquamation, muscle pain and hypertension during the following days. We described this clinical presentation as idiosyncratic hypersensitivity due to mercury intoxication.

Elemental mercury is used in manufacturing and industrial processes (mining, smelting), household, medical and electrical devices (for example, thermometers, thermostats, electrical switches, dental amalgam), and in folk remedies. Thus, environmental release of elemental mercury that results in human exposure can occur in many different locations [8]. Sarıkaya *et al.* reported a case of acute elemental mercury poisoning that they followed presenting with abdominal pain, diarrhea and high fever. The patient’s daughter had brought mercury home from school and heated it. One day after the event, the patient’s 14-month-old daughter died of mercury poisoning [9]. Seen from this aspect, mercury should be kept under safer conditions and whether it should continue to be held in schools should be discussed.

Rowens *et al.* reported that four people from the same family died as a result of respiratory failure caused by heated elemental mercury [10]. Glezos *et al.* reported a patient that was hospitalized and followed up for seven days because of high fever, pneumonia, generalized maculopapular rash, and axillary lymphadenopathy, and it was learned one week after discharge that he had been present in an environment in which mercury had been heated for three hours [11]. Bamonti *et al.* reported that cracked mercury dental amalgam was a possible cause of fever of unknown origin [12]. The patients do not realize that they have been exposed to the mercury vapor because it is colorless and odorless. Therefore, patients with such symptoms should be questioned about possible exposure to mercury.

## Conclusions

Environmental release of elemental mercury that results in human exposure can occur in many different locations such as schools, universities, homes, healthcare facilities, public utilities and manufacturing facilities. Thus, although it is rarely seen, patients should be questioned about being exposed to mercury or other metals when a case of fever of unknown origin is encountered.

## Consent

Written informed consent was obtained from the patients for publication of this manuscript and any accompanying images. A copy of the written consent is available for review by the Editor-in-Chief of this journal.

## Abbreviations

CRP: C-reactive protein; Cd: cadmium; °C: Centigrade; CT: computed tomography; CMV: *Cytomegalovirus*; EBV: *Epstein*-*Barr virus*; ESR: erythrocyte sedimentation rate; FUO: fever of unknown origin; Hg: mercury; Ig: immunoglobulin; ICP-MS: inductively coupled lasma - mass spectrometer; LDH: lactate dehydrogenase; Mn: manganese; RBC: red blood cell; TA: tension arterielle; USG: ultrasonography; WBC: white blood cell; ZnCl2: zinc chloride.

## Competing interests

The authors declare that they have no competing interests.

## Authors’ contributions

GCS was involved in the conception of the report, literature review, manuscript preparation, editing and submission. FAA, AUK, YG, ET and IS were responsible for the manuscript critique and review. All authors have read and approved the final manuscript.
